# The NEOtrap – en route with a new single-molecule technique

**DOI:** 10.1016/j.isci.2021.103007

**Published:** 2021-09-25

**Authors:** Sonja Schmid, Cees Dekker

**Affiliations:** 1 Nanodynamics Lab, Laboratory of Biophysics, Wageningen University, Stippeneng 4, 6708WE Wageningen, the Netherlands; 2 Department of Bionanoscience, Kavli Institute of Nanoscience, Delft University of Technology, Van der Maasweg 9, 2629 HZ Delft, the Netherlands

**Keywords:** Physical chemistry, Nanotechnology, Protein

## Abstract

This paper provides a perspective on potential applications of a new single-molecule technique, viz., the nanopore electro-osmotic trap (NEOtrap). This solid-state nanopore-based method uses locally induced electro-osmosis to form a hydrodynamic trap for single molecules. Ionic current recordings allow one to study an unlabeled protein or nanoparticle of arbitrary charge that can be held in the nanopore's most sensitive region for very long times. After motivating the need for improved single-molecule technologies, we sketch various possible technical extensions and combinations of the NEOtrap. We lay out diverse applications in biosensing, enzymology, protein folding, protein dynamics, fingerprinting of proteins, detecting post-translational modifications, and all that at the level of single proteins – illustrating the unique versatility and potential of the NEOtrap.

## Graphical abstract

**Figure:**
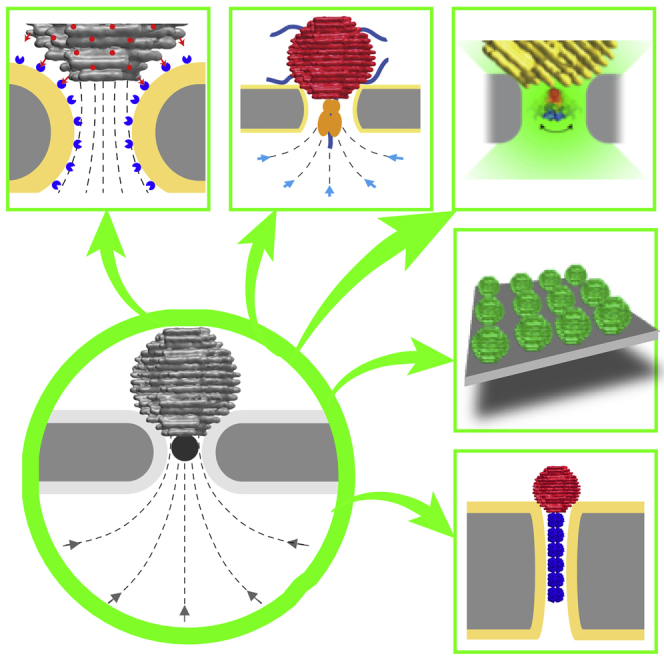

## Teaser

Nanopores; Single-molecule detection; Physical chemistry; Nanotechnology; Protein

## Introduction

Proteins are involved in all vital processes in our cells (Berg et al., 2002). Their precise 3-dimensional structures are increasingly becoming available through recent advances in electron microscopy and also from x-ray crystal diffraction, NMR, and other techniques (Berman et al., 2020; Cheng, 2018; Dobson, 2019). Yet, such spatial information from time-frozen snapshots cannot reveal the energies that drive the vital protein functional cycles, involving conformational dynamics as well as transient protein–protein interactions and diverse cofactor and nucleotide interactions (Henzler-Wildman et al., 2007; Lerner et al., 2018a). Clearly, additional time domain information is needed. It is, however, often difficult to measure protein kinetics experimentally. A challenge is that dynamics normally occur in complex nonsynchronized ensembles, leading to ensemble-averaged time constants that cannot reveal the molecular dynamics at the single-molecule level. For the experimentalist, there are generally two options to make the decisive time information detectable: either one aims for ensemble synchronization or for single-molecule experiments.

In the first case, which is widely used throughout the life sciences, the experimentalist finds a way to artificially bring the ensemble out of equilibrium, which then allows one to watch and quantify within which time the entire ensemble relaxes back to equilibrium (Dill and Bromberg, 2010). This can, for example, be done by adding, at “time zero,” a quantity of substrate molecules such as ATP, a substrate protein, a cofactor, etc., that is necessary for the protein reaction under study. As a result, the concerted molecular process can be observed at the ensemble level. There are however fundamental limitations to this approach, for example, (i) it is only applicable to a subset of molecular processes that do occur out of equilibrium; (ii) often the experimentally recorded signal is the result of a complex sequence of stochastic molecular processes that can still not be disentangled as one rate-limiting step overrules everything, and (iii) the molecular mechanism remains unknown. We note that in the exceptional case of a plain two-state system, correlation-based fluctuation analysis can resolve the two transition rates. This is however not generalizable to three and more states, where at best state life times can be resolved, but generally not specific transition rates, thus rendering the full kinetic connectivity of states inaccessible in general ensemble experiments.

In the second case, applying single-molecule technology (Banerjee et al., 2021; Hellenkamp et al., 2018; Lerner et al., 2018a; Miller et al., 2017; Mohapatra et al., 2020), the detection method is tuned to such a high sensitivity that it allows one to observe the time evolution of just one single molecule along a certain reaction coordinate – in this way overcoming the need for ensemble synchronization. As a result, single-molecule techniques can be applied more broadly to investigate equilibrium and nonequilibrium processes (including nonequilibrium steady-state processes) (Dill and Bromberg, 2010; Juette et al., 2016; Schmid et al., 2016). The price for these added opportunities is usually paid in the form of quite sophisticated instrumentation. Common prominent techniques that have proven themselves in biophysical studies are fluorescence and force spectroscopies, scanning probe microscopies, nanopore technologies, and newer techniques such as interferometric scattering (Kukura et al., 2009; Soltermann et al., 2020). Single-molecule techniques were used to map out, in great mechanistic detail, many molecular motor proteins such as the rotary motor F_0_F_1_-ATPase (Noji et al., 1997; Yasuda et al., 2001), linear motors such as kinesin (Bornschlögl et al., 2009; Helenius et al., 2006; Svoboda et al., 1993; Vale et al., 1996) and myosin (Forkey et al., 2003; Mehta et al., 1999; Yildiz et al., 2003), and more recently structural maintenance of chromosomes (SMC)-driven DNA loop extrusion (Ganji et al., 2018; Kim et al., 2020). Also, equilibrium fluctuations could be resolved and quantified in biomolecular systems of diverse scale and composition (Hellenkamp et al., 2017; Kilic et al., 2018; Lerner et al., 2018b; Schmid and Hugel, 2020; Wang et al., 2021). High-speed atomic force microscopy (AFM), which provides 2-dimensional spatial information at video-rate, was used to record movies of internal conformational changes, for example, a myosin walking along actin (Kodera et al., 2010) or an SMC protein complex making a scrunching motion (Ryu et al., 2020). Magnetic and optical tweezers have been widely used to examine DNA-processing proteins such as polymerases (Hodges et al., 2009) or helicases (Cheng et al., 2011; Manosas et al., 2012; Stanley et al., 2006). Nanopores have been used for a wide variety of biophysics studies (Galenkamp et al., 2020; Rozevsky et al., 2020; Schmid et al., 2021; Schmid and Dekker, 2021; Yusko et al., 2012) and applied to develop DNA sequencing (Lieberman et al., 2010; Manrao et al., 2012) at the single molecule level.

In many of these cases, the observation of a sequence of states made the “inner workings” of the protein visible and allowed one to relate molecular processes with experimentally determined energies, to distinguish energetically driven processes from thermal fluctuations, and to uncover the crucial structural elements (down to atomic scale through mutations). Single-molecule techniques have provided major progress in understanding proteins. In a way, they provide mechanistic insights that lift our knowledge on protein systems beyond the biochemical “blobology” cartoon level. Suddenly, energies, forces, duty cycles, etc. can be quantified from experiments – providing key input knowledge for further bioengineering.

Yet, single-molecule techniques also face limitations. In fluorescence-based single-molecule techniques, photobleaching is a central restriction. While, in principle, single-photon counting offers a time resolution in the nanosecond range and technically fluorescence experiments can last for hours, photobleaching limits the temporal bandwidth of any given experiment to three orders of magnitude or less (Lu et al., 2019; Schmid and Hugel, 2020; Zosel et al., 2018). Other limitations of single-molecule techniques come, for example, from surface immobilization, in particular for AFM which is surface based by necessity, or by the presence of artificial labels in fluorescence-based experiments where dyes are attached covalently following site-specific mutagenesis. Force spectroscopies (AFM, optical or magnetic tweezers, or acoustic force spectroscopy (Sitters et al., 2015) also make use of artificial “handles” and apply mechanical force that can distort the protein's native state up to unfolding.

Electrical nanopore detection, where a single protein is studied based on how it modulates the ionic through-pore current, requires a considerable ionic strength, but this has not prevented the use of notoriously ion-sensitive DNA-processing proteins in commercialized nanopore-based DNA sequencing (Loman and Watson, 2015). Notably, nanopore detection provides a number of advantages over the other techniques: it is very sensitive and can for example discriminate the presence or absence of single methyl groups (Clarke et al., 2009; Tourancheau et al., 2021) and even different enantiomers of the same compound (Kang et al., 2006), all without the need for artificial labels or other modifications, it is affordable, and it features a vast electrical bandwidth from μs to hours. The broad temporal bandwidth is particularly attractive given the broad range dynamics of proteins where one likes to resolve both fast fluctuations and rarer conformational transitions and interactions. Hence, nanopore technology is in many ways ideal to study protein dynamics. However, until recently, the Achilles heel of solid-state nanopore detection (Xue et al., 2020) was the prohibitively fast translocation of folded proteins. This limitation was largely solved by the recent invention of the nanopore electro-osmotic trap, NEOtrap (Schmid et al., 2021).

## The NEOtrap, current status and future technical extensions

**Figure 1:**
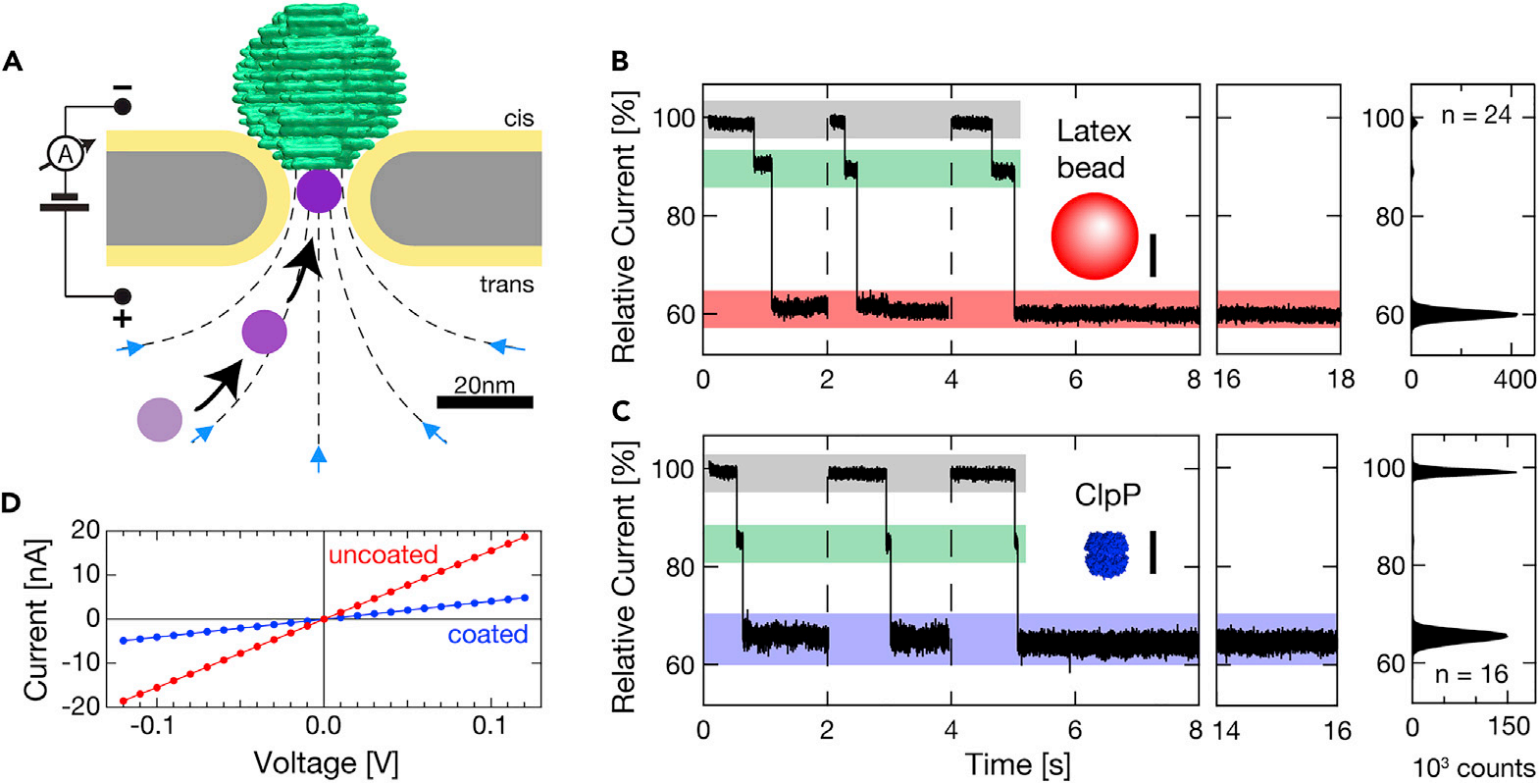
The Nanopore-Electro-Osmotic trap (NEOtrap) (A) Illustration of the passivated solid-state nanopore (gray) with a docked DNA-origami sphere (green) and a single molecule (purple) being trapped by hydro-dynamic flow (blue arrows and dashed lines). (B) Current traces (5 kHz sampling) showing open-pore current (gray overlay), DNA-origami docking (green), and latex bead trapping (red, 0.004% (w/v) 20 nm latex beads FisherScientific, Landsmeer, NL) in a 29.5 nm diameter SiN nanopore. Scale bar 10 nm. Constant and uniform trapping lasted >10 s, leading to sharp peaks in the multitrace histograms on the right. Gauss fitting yields the three peak positions and standard deviations: 99.0 ± 0.6%, 89.0 ± 0.8%, 60.0 ± 0.9%. (C) Same as B but with protein ClpP trapping (blue, pdb: 1yg6, 10 nM) using a 23.5 nm-diameter SiN nanopore. Gauss fitting of the histogram yields the three peak positions and standard deviations: 99.0 ± 0.6%, 85.0 ± 0.9%, 65.4 ± 1.2%. NEOtrap measurements were performed in 600 mM KCl, 50 mM HEPES, 5 mM MgCl_2_, pH7.5 using a lipid bilayer coated (Venkatesan et al., 2011; Yusko et al, 2011, 2016) silicon nitride nanopore as detailed previously (Schmid et al., 2021). (D) Corresponding current vs. voltage curves of the 29.5-nm-diameter pore used in (B), uncoated and coated as specified. DNA-origami image used with permission of RSC, from Ref (Kopatz et al., 2019); permission conveyed through Copyright Clearance Center, Inc.

The NEOtrap is a cavity for a single protein that is formed by electrophoretically docking a charged object, like a DNA-origami cork, onto a passivated solid-state nanopore (Figure 1). Such a docking event has two effects: (i) a nanocavity of desired nanometric dimensions, ∼(10nm)^3^, is formed whose size can be chosen to fit a desired protein of interest and (ii) the presence of the highly charged DNA origami in the electric field causes its counter ions to move directionally along the field lines, thus inducing a strong electro-osmotic water flow. The latter creates a hydrodynamic trapping potential on the order of 10–20 k_B_T (Schmid et al., 2021), which can trap a protein irrespective of its charge. Once a protein is trapped by locating it into the NEOtrap, it can be sensed at the most sensitive region of the nanopore for extended times up to many hours (Schmid et al., 2021). In this way, the NEOtrap improves the observation time of unmodified proteins of any charge by a factor of one million to one billion, as compared to the brief time that free translocation takes. Notably, the NEOtrap uses electro-osmotic flow to trap analytes inside a solid-state nanopore, in contrast to earlier work where, for example, nanoparticles were docked onto solid-state nanopores with smaller diameter, thus preventing translocation due to size constraints (Tsutsui et al., 2013). The NEOtrap blocking current induced by the presence of the protein shows a linear size dependence on protein size for roughly globular proteins, whereas shape dependence is observed for nonglobular proteins. Furthermore, even different conformational states could be resolved within one protein. Specifically, conformational differences induced by the presence or absence of just one phosphate group in a nucleotide could be discriminated. These first data prove the sensitivity of the NEOtrap which can now be harnessed for many more studies of a large variety of protein systems.

**Figure 2:**
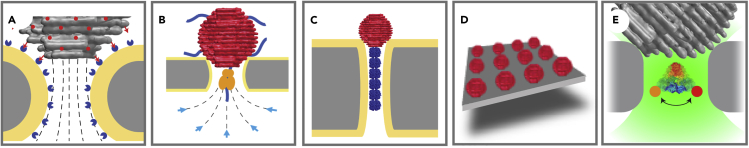
The NEOtrap and envisioned technical extensions (A) New opportunities arise through chemical functionalization of the pore passivation layer and the origami sphere to lock it in place. (B) Attachment of affinity baits such as specific DNA or RNA motives, aptamers, affimers, or peptide tags. (C) Pore shapes customized for the system under study. (D) Pore arrays for high-throughput single-molecule studies (not to scale). (E) Electro-optical combination with fluorescence/FRET detection. DNA-origami image used with permission of RSC, from Ref (Kopatz et al., 2019); permission conveyed through Copyright Clearance Center, Inc.

In this paper, we provide a brief outlook on possible extensions and applications of the NEOtrap. This new single-molecule technique can be expanded in many ways, as illustrated in Figure 2. Surface functionalization of the DNA-origami sphere and the passivation coating on the nanopore offer many possibilities (Figure 2A). It should, for example, be possible to dock the origami sphere irreversibly onto the lipid-bilayer passivation layer by using covalently attached lipid anchors (cholesterols, porphyrins). This would reduce thermal fluctuations of the sphere, leading to better trapping of even smaller proteins or peptides, which currently escape the trap relatively quickly. The trapping potential can be further tuned by means of net-negative or net-positive passivation layers that will enhance or reduce the electro-osmotic water flow, respectively. This can be achieved by adding lipids with negatively or positively charged head groups to the passivating lipid bilayer coating or by changing the solution pH (Firnkes et al., 2010; Fujinami Tanimoto et al., 2021), but the latter may have undesirable effects on the analyte. Alternative passivation protocols, such as tethered lipid bilayers (Andersson and Knoll, 2019) or grafted polymers (Awasthi et al., 2020; Giamblanco et al., 2018b; Roman et al., 2017), may improve the coating stability and tolerate higher voltages without adding noise.

The origami sphere can also be functionalized to become analyte specific (Figure 2B), which opens new applications. Specific binders, such as protein-based or other ligands, nanobodies, affimers, aptamers, etc., can be added to the outside of the sphere by simple hybridization of DNA oligos. An obvious application will be to “catch” analytes from the bulk solution which subsequently are interrogated in the NEOtrap – allowing for sensitive biosensing. To study DNA-interacting proteins such as polymerases or CRISPR-Cas systems, the origami sphere can be functionalized with the required (specific) nucleic acid sequence, at defined positions and numbers. Furthermore, the design of the origami structure itself can be modified to different shapes. However, we found that a spherical shape caused the most reproducible current blockade signals upon docking (i.e. NEOtrap formation), which tremendously simplifies data interpretation and therefore provides a decisive advantage over other designs. A different sphere material could be chosen, such as, for example, agarose beads, or other charged and permeable materials capable of inducing electro-osmosis (Li et al., 2020). However, the interparticle homogeneity (size, shape, density) remains an important selection criterion.

Different shapes of the nanopore sensor can be used to achieve optimal sensitivity for a given protein effect (Figure 2C). For example, longer nanopore channels in thick silicon nitride (SiN) (Niedzwiecki et al., 2020) or polymer membranes (Buchsbaum et al., 2014) provide for a long cavity that is well suited to test, for example, protein fibrillization in a time-resolved way, as further discussed below. The docking position for the origami sphere can also be engineered, for example, with a cup-shaped nanopore opening, to further control the sphere position.

The current one-nanopore scheme can be expanded to nanopore arrays to examine many proteins in parallel (Figure 2D). Interestingly, trapping of proteins in the NEOtrap is a self-limiting process where, for a well-chosen pores size, a protein will only be captured if the trap is not already occupied by a protein (Schmid et al., 2021), facilitating NEOtrap arrays with uniform single-protein occupation. The NEOtrap array then serves as the single-molecule version of industrial multiwell plates, which can easily be refreshed with new single molecules or particles by simple voltage inversion. The readout of many NEOtraps in parallel can be achieved with electrode arrays (such as in the MinION system (Loman and Watson, 2015) or using optical detection (Sawafta et al., 2014), similar to strategies pursued with protein nanopore arrays (Huang et al., 2015). In fact, even without actually reading out the traps, NEOtrap arrays can serve as a mechanism to easily localize single molecules or nanoparticles for a range of other high-throughput experiments. Alternatively, or in addition to this parallelization in arrays, high throughput can be achieved by sequential trapping in automated time series using voltage protocols as desired. Both approaches enable high-throughput screening at the single-molecule level.

In addition to purely electrical detection, the ionic current recording can be conveniently combined with an orthogonal optical readout (Keyser et al., 2005) such as fluorescence (Cai et al., 2019; Ivankin et al., 2014; Wang et al., 2018) or FRET (Hemmig et al., 2018) (Figure 2E). In this way, the NEOtrap current recordings can be *in situ* calibrated with nm-sensitive FRET signals. Such NEO-FRET would benefit from the best of both worlds: a broad temporal bandwidth via electrical detection and signal interpretation with sub-nm-precision from the correlated optical detection. The electro-optical combination of nanopores with zero-mode waveguides has been used already to further localize the confocal laser excitation (Assad et al., 2017; Jadhav et al., 2019; Klughammer and Dekker, 2021). And, additional spectroscopic combinations such as vibrational spectroscopy, circular dichroism, etc. may also be possible.

It may also be useful to discuss some of the limits of the NEOtrap. Concerning kinetics, the attainable trapping speeds can be tuned by the concentration of the DNA-origami sphere and the analyte. For the analyte's escape rate, a near-exponential decrease with molecular weight was found, resulting in seconds-to-hours-long trapping for 45-kDa or 360-kDa proteins, respectively (Schmid et al., 2021). Trap release happens essentially instantaneously upon voltage inversion with immediate undocking of the DNA-origami sphere. The NEOtrap's signal-to-noise depends largely on the studied effect and desired bandwidth. For the data shown in Figure 1, the signal-to-noise ratio amounts to 34 and 23 for bead and protein trapping, respectively (defined as ΔI/σ of the multitrace histogram obtained using 5 kHz sampling). The NEOtrap's noise level is limited by fluctuations of the DNA-origami, coating instabilities, and the usual nanopore noise sources including 1/f flicker noise, thermal, dielectric, and capacitive noise (Fragasso et al., 2020). Overall, a mass resolution of ca. 20 kDa was found for globular proteins (Schmid et al., 2021).

## A wide range of applications of the NEOtrap

**Figure 3:**
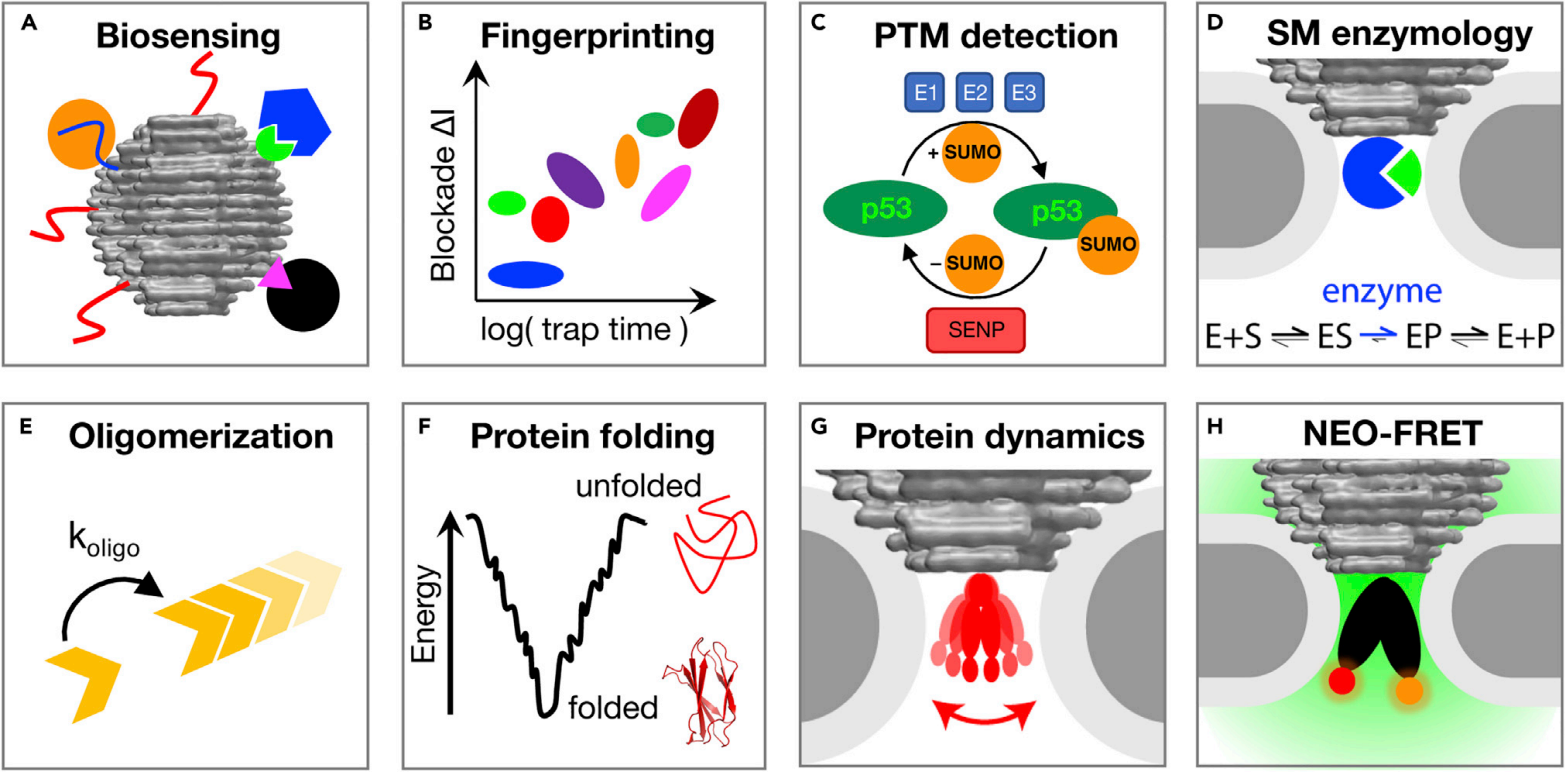
Envisioned applications of the NEOtrap (A) Affinity-based biosensing in mixtures. (B) Fingerprinting proteins based on mass and shape. (C) Detecting post-translational modifications (PTM) such as ubiquitination or glycosylation. (D) Single-molecule (SM) enzymology. (E) Real-time observation of protein oligomerization kinetics. (F) Chemical or thermal single-protein unfolding/refolding kinetics. (G) Conformational dynamics within a single protein. (H) Nanopore electro-osmotic FRET (NEO-FRET) for correlative electro-optical studies. DNA-origami image used with permission of RSC, from (Kopatz et al., 2019); permission conveyed through Copyright Clearance Center, Inc.

With the NEOtrap at hand, many biophysics experiments now become possible that were previously inaccessible, for example, by the short observation time of proteins using nanopore detection. On a general level, biosensing –that is, the detection of a certain biomolecule or metabolite – is a huge branch of applied research at the intersection of solid-state physics, biochemistry, electrochemistry, and engineering (Akkilic et al., 2020). Specifically functionalized NEOtraps may achieve such analyte detection with single-molecule resolution (Figure 3A). By employing affinity tags on the DNA-origami spheres and a voltage stepping scheme, high-throughput screening for analytes should be possible with the NEOtrap. Already without any further modifications, the NEOtrap can be used to read the size- and shape-dependent signals of thousands of proteins. A library of fingerprints can be collected (Figure 3B) and used as a training set for pattern recognition algorithms to facilitate the label-free identification of protein mixtures. Such machine-learning-enhanced sensing approaches (Arima et al., 2018; Cao et al., 2020; Meyer et al., 2020) may provide a basis for single-cell proteomics in the future. In all this, the label-free aspect is a key advantage of the NEOtrap as that provides the option of direct measurements of scarce and nonpurified biological samples, such as lysate, blood, sweat, saliva (Galenkamp et al., 2018; Sze et al., 2017) without additional preprocessing. The ease of integration into small portable devices is yet another advantage over other techniques (Loman and Watson, 2015).

Native proteins are often post-translationally modified (Figure 3C) where specific proteinaceous, sugar, or other groups are attached to amino acids (Lerner et al., 2018a). NEOtrap sensing may be used to detect such post-translation modifications (PTMs). These include for example (de-)ubiquitination, SUMOylation, neddylation, pupylation, ISGylation, polysaccharides, and possibly thiol-bridge formation (Ribet and Cossart, 2018). Smaller PTMs such as phosphorylations, nitrosylation, small sugars, etc. may be detectable directly as they induce conformational rearrangements (Humphrey et al., 2015) or can be made observable by specific labeling with bulky tags. By detecting the time-dependent product buildup during enzymatic reactions, the NEOtrap offers a new single-molecule readout for enzyme assays such as post-translational additions, proteolytic cleavage, or protein splicing (Kane et al., 1990). In comparison to regular translocation experiments (Fennouri et al., 2013; Giamblanco et al., 2018a; Karawdeniya et al., 2018; Kasianowicz et al., 1996; Ma et al., 2019; Zhao et al., 2009), the NEOtrap provides much longer observation times for analytes with diverse net charges, thus leading to a tremendously increased information gain per each single analyte molecule.

This can be generalized to single-molecule enzymology (Figure 3D), where conformational changes of an enzyme during the catalytic conversion of a substrate to a product are followed using the NEOtrap. Protein–protein interactions can also be studied. Given that the electro-osmotic capture of the NEOtrap is mass independent, protein affinities can be determined simply by counting the number of bound and unbound species that are trapped from solution, one by one. Furthermore, the early onset of protein oligomerization can be detected in a label-free way (Figure 2E). Oligomer nucleation is the crucial and rate-limiting step toward protein filament formation, which is, for example, a key determinant of neurodegenerative diseases. Interestingly, it is precisely the growth kinetics of these early onset oligomers – that are understood to be the toxic species in Alzheimer's, Parkinson's, Huntington's disease etc.(Ingelsson, 2016; Liu et al., 2015) – that are difficult to detect experimentally.

Single-protein folding experiments make for yet another possible application of the NEOtrap (Figure 3F). Given that DNA-origami is surprisingly robust towards denaturants (Ramakrishnan et al., 2016), diverse chemical unfolding conditions can be screened to determine reversible unfolding and refolding rate constants on one single protein, plus corresponding energies, chevron plots, etc., which provides unique insight into protein structural stabilities with direct implications on protein function. In addition, heat-induced unfolding can be studied, for example, using local laser heating. Beyond equilibrium unfolding and refolding, this also enables temperature-jump experiments to probe single-protein folding out of equilibrium. Notably, while folding and unfolding have been studied at a bulk population level (Oukhaled et al., 2007; Payet et al., 2012), with the NEOtrap, chemical and thermal unfolding and refolding may be studied at the level of one single trapped molecule, similar to mechanical unfolding using force spectroscopies (Woodside and Block, 2014).

Finally, the NEOtrap will also allow to address the holy grail of single-molecule techniques, that is, resolving the intrinsic conformational dynamics of a single protein (Figure 3G). In our first study of the NEOtrap, we already provided proof-of-principle data that showed conformational sensitivity, clearly suggesting that transitions between individual conformations may be detected under optimal conditions such as a further increased trapping times, using lipid-anchored origami structures, or slightly larger proteins. It will be interesting to see what the limits are for resolving conformational transitions with the NEOtrap, which – unlike FRET – is not limited to one specific reaction coordinate. Indeed, nanopore detection has already revealed hundreds of catalytic cycles of a single enzyme that were undetectable by FRET (Galenkamp and Maglia, 2020). The label-free study of diverse dynamic protein systems in the range of microseconds to minutes bears the potential to reveal hierarchical correlations between fast and slow transitions that were missed with existing techniques that sample either the fast or the slow end of the time axis.

Entirely new experiments may become possible using the NEO-FRET combination (Figure 3H), where electrical and optical observations can be correlated in an experimentally orthogonal way. Such an additional dimension of information can, for example, be used to correlate the presence of a fluorescently labeled nucleotide with electrically measured protein dynamics, in real time.

## Outlook

This perspective discussed many new experimental routes that become possible with the NEOtrap. Electro-osmotic trapping is a very versatile way to trap single proteins and other nanoparticles hydrodynamically, which works for a wide range of positively and negatively charged particles, without extra labeling or other modifications. We discussed a range of strategies to further expand the NEOtrap beyond the current design, such as chemical modifications of the pore coating or the electro-osmotically active sphere, to tune the trap characteristics as desired. We described biotechnological functionalizations to attain analyte specificity, different chip designs that offer single-molecule well plates or adaptations to the protein system of interest, as well as combined detection schemes for the NEOtrap such as electro-optical combinations.

This wealth of new technical developments offers many opportunities to address current needs in biotechnology and protein science, ranging from specific biosensing, via protein fingerprinting and single-molecule proteomics, to the time-resolved detection of PTM's in small, unmodified samples. Protein–protein interactions can be studied, and chemical or thermal unfolding-refolding experiments become possible – all at the single-molecule level. Single-molecule enzymology, or more broadly, protein conformational dynamics can now be explored electrically without artificial protein modifications. We anticipate that the NEOtrap can contribute to the elucidation of diverse dynamic nanoscale processes in proteins and beyond.

Using the advances discussed herein, we aim to develop the NEOtrap into a label-free protein dynamics detector that excels at the single-molecule level, and we warmly invite all members of the nanopore community to join our travels into the unexplored nanopore territory of the inner workings of proteins.

## Data Availability

The source data is available at https://doi.org/10.5281/zenodo.5270451.
